# Combined Therapy with microRNA-Expressing *Salmonella* and Irradiation in Melanoma

**DOI:** 10.3390/microorganisms9112408

**Published:** 2021-11-22

**Authors:** Wonsuck Yoon, Yongsung Park, Seunghyun Kim, Yongkeun Park, Chul Yong Kim

**Affiliations:** 1 Allergy Immunology Center, College of Medicine, Korea University, Seoul 02841, Korea; seunghyun@korea.ac.kr; 2 Department of Life Science and Biotechnology, Korea University, Seoul 02841, Korea; dcomtrue@korea.ac.kr (Y.P.); actsbio@naver.com (Y.P.); 3 Department of Radiation Oncology, College of Medicine, Korea University, Seoul 02841, Korea

**Keywords:** melanoma, bacterial treatment, *Salmonella* *Typhimurium*, radiation therapy

## Abstract

Anticancer treatment strategies using bacteria as a vector are currently expanding with the development of anticancer drugs. Here, we present a research strategy to develop anticancer drugs using bacteria that contain miRNAs. We also present a strategy for the development of novel bacterial anticancer drugs in combination with radiation. *Salmonella* strains expressing miRNA were produced by modifying the miRNA expression vector encoding INHA, a radiation-resistant gene developed previously. The anticancer effect of INHA was confirmed using skin cancer cell lines. We also tested a combination strategy comprising bacteria and radiation for its anticancer efficacy against radiation-resistant mouse melanoma to increase the efficacy of radiation therapy as a novel strategy. The recombinant strain was confirmed to promote effective cell death even when combined with radiation therapy, which exerts its cytotoxicity by enhancing reactive oxygen species production. Moreover, a combination of bacterial and radiation therapy enhanced radiotherapy efficacy. When combined with radiation therapy, bacterial therapy exhibited effective anti-cancer properties even when administered to animals harboring radiation-resistant tumors. This strategy may promote the secretion of cytokines in cells and more effectively reduce the number of bacteria remaining in the animal. Thus, this study may lead to the development of a strategy to improve the effectiveness of radiation therapy using *Salmonella* expressing cancer-specific miRNA for intractable cancers such as those resistant to radiation.

## 1. Introduction

Over the past two decades, many studies have shown that *Salmonella* and tumor microenvironments are closely connected with respect to hypoxia [1]. *Salmonella* preferentially accumulates in tumors of the liver at a ratio of 1000:1, and the rapid growth of tumors results in low oxygen, low pH, and necrosis [2,3,4]. Using these characteristics of *Salmonella* and tumor microenvironments, various *Salmonella* strains or those carrying a variety of effector genes have been developed and examined [5,6]. In addition, we have at-tempted to regulate target cancer-associated genes by expressing miRNAs in a *Salmonella* strain [5,7,8,9,10,11].

It was confirmed that colon cancer and skin cancer were suppressed by miRNAs facilitating the expression of inhibin alpha [12,13]. This represents a new strategy, and vectors expressing miRNAs have been delivered through cancer-specific bacteria. The inhibin gene is also overexpressed in radiation-resistant skin cancer [14,15]; thus, it is necessary to analyze the correlation between radiation therapy and these genes.

INHA, the gene encoding the inhibin alpha subunit, is involved in mammalian folliculogenesis. The downregulation of INHA caused an increase in apoptosis and induced ROS production via a mitochondrial pathway in the cell [16]. On the other hand, it is shown that normal cells continuously generate ROS as a result of electron leakage from mitochondria through the electron transport system. For the maintenance of the intracellular redox balance, these toxic ROS are instantly removed by an endogenous antioxidant defense system. It was also reported that the ROS level of cancer cells is already high, reaching the threshold for tolerating ROS [17,18], and the irradiation induces the generation of ROS via an ionizing exposure. These means that the ROS production by INHA miRNA and irradiation could play some roles in eradicating the cancer cells, and it could overcome the limitation of radiation therapy that the radiation depends on the tumor size.

Radiation therapy is currently administered to 40–50% of all cancer patients as the initial treatment, but the frequency of resistance to treatment is increasing due to the expression of radiation resistance genes [19,20]. Various radiation-sensitizing agents have been developed [21,22], and the effects of intestinal bacteria on radiation sensitivity have been recently reported [23,24]. This raises expectations for the development of strategies for pretreatment that use radiation-sensitive drugs and bacteria.

This study aimed to develop a system to test the possibility of enhancing the efficacy of radiotherapy by delivering a *Salmonella* strain that expresses radiation-sensitizing miRNA for more effective delivery to cancer cells and to apply it to radiation-resistant skin cancer cells.

## 2. Materials and Methods

### 2.1. Construction of Attenuated Salmonella Strains Expressing miRNA

For this study, we used the *S. Typhimurium* BRD509 strain, which is an aroA aroD mutant of strain SL1344. Expression vectors encoding miRNA against INHA and a scrambled miRNA (mi-Cont) were transformed into *S. Typhimurium* SF586, which was used to maintain the INHA plasmid to increase the transformation efficacy in the BRD509 strain. The plasmid isolated from the *Salmonella* SF509 strain was used to construct the INHA plasmid-harboring BRD509 strain. Vector-based siRNAs targeting human INHA or mouse INHA were derived from the mRNA sequences of human (GenBank: NM_002191.3) or mouse INHA (GenBank: NM_001329843.1). To construct the plasmids expressing human or mouse INHA siRNAs, we used a modified BLOCK-iT Pol II miR RNAi expression vector system (Invitrogen, Carlsbad, CA, USA), in accordance with the manufacturer’s protocol. The INHA miRNA-coding and mock plasmids were prepared such that the CMV promoter was replaced with the *Salmonella*-operated T7 promoter. The various strains were then constructed using the plasmids harboring INHA miRNA or scrambled miRNA.

### 2.2. Transfection of Salmonella into Cells and Radiation

Mouse melanoma B16F10 cells were cultured in Dulbecco’s modified Eagle’s media (DMEM) with 10% fetal bovine serum (FBS), (Hyclone, Korea) supplemented with the following antibiotics: 100 units/mL penicillin and 100 mg/mL streptomycin (Sigma, St. Louis, MO, USA). The melanoma cell line used in this study was distributed and used by an accredited Korean cell line bank, and additionally, a human melanoma cell line (A375) was also tested. Each B16F10 cell was seeded at a density of 2 × 10^5^ cells in a 60-mm cell culture dish and incubated overnight at 37 °C in a 5% CO_2_ incubator, so that the cells reached 85–90% confluence. *S. Typhimurium* expressing miRNA plasmids was infected at a multiplicity of infection (MOI) of 1:1000 (B16F10 cells: *Salmonella*), and cells were irradiated with 8 Gy, after which 100 μg/mL gentamycin (Invitrogen, Carlsbad, CA, USA) was added. Twenty-four hours after incubation, the cells were analyzed.

Irradiation was carried out and the management of the radiation equipment for animals and cells was carried out according to the manufacturer’s guidelines.

### 2.3. Salmonella Invasion Assay

Mouse melanoma cells were infected with *S. Typhimurium* at an MOI of 500 for 1 h at 37 °C. To quantify internalized *S. Typhimurium*, extracellular *S. Typhimurium* were killed by incubating with 100 μg/mL gentamycin for 1 h. After antibiotic treatment, the cells were washed again with PBS to remove residual gentamycin and then lysed with 1% Triton X-100 for 5 min at 37 °C. Intracellular *S. Typhimurium* was quantified by plating 10-fold serial dilutions of cell lysates on LB plates without antibiotics [25,26].

### 2.4. LDH Assay

Following treatment with siRNAs or engineered *S. Typhimurium*, the viability of B16F10 melanoma cells was assessed using a trypan blue dye exclusion assay and lactate dehydrogenase (LDH) assay. The exclusion of trypan blue dye by viable cells was evaluated within the grid of a hemocytometer. Cell survival rate was calculated as the number of viable cells divided by the total number of cells. A minimum of 200 cells were counted. To evaluate plasma membrane damage, LDH assays were performed by measuring the release of LDH from the cytosol into the culture medium, using a CytoTox 96 non-radioactive Cytotoxicity Assay kit (Promega, Madison, WI, USA) in accordance with the manufacturer’s protocols. LDH activity was determined by measuring the conversion of a tetrazolium salt into a red formazan product using an ELISA reader at 492 nm. Treatment-induced LDH release was calculated as a percentage of the total LDH activity found in the culture medium.

### 2.5. Protein Extraction and Immunoblot Analysis

Reagents for Western blotting were obtained from Bio-Rad (Hercules, CA, USA). Proteins were electrophoretically separated on a sodium dodecyl sulfate (SDS)-polyacrylamide gel, followed by transfer to nitrocellulose membranes. The transformation of pcDNA6.2-GW/EmGFP-mi-INHA into *S. Typhimurium* was assessed by monitoring the co-cistronic expression of EmGFP using a monoclonal anti-GFP antibody (ab1218, Abcam). The expression of bacterial chaperone protein DnaK was probed for simultaneously using an anti-DnaK antibody (ab69617, Abcam) as the internal loading control. The other proteins in mammalian cancer cells were detected using the following primary anti-bodies: INHA (ab81234) and Bcl-2 (ab59348) from Abcam; Bcl-xL (2764) and actin (4967) from Cell Signaling (Beverly, MA, USA). Secondary antibodies were horseradish peroxidase labeled anti-mouse or anti-rabbit antibodies. Binding was detected by chemiluminescence signals, according to the manufacturer’s instructions (Roche, Basel, Switzerland).

### 2.6. Real-Time Quantitative PCR Analysis

The total RNA from *Salmonella* strains transformed with INHA and scrambled plasmids was extracted for RT-qPCR. Reverse transcription was performed using the iScript cDNA synthesis kit (Bio-Rad) according to the manufacturer’s protocol. The RT cDNA reaction products were subjected to quantitative real-time PCR using CTFX 96 Real-Time System (Bio-Rad) and SYBR Green Supermix (Bio-Rad) according to the manufacturer’s protocol. All expression levels were normalized to DnaA levels of the same sample. Percent expression was calculated as the ratio of the normalized value of each sample to that of the corresponding untreated control cells. All real-time PCRs were performed in triplicate. Optimal primer sequences were designed using Primer-BLAST: qPCR primer, 5′-TCCCAAGCCATCCTTTTCCCAG-3′ and 5′-TCACCTGGCGGCTGCGTGTAT-3′.

### 2.7. Reactive Oxygen Species (ROS) Assay

Intracellular hydroperoxide and oxidative stress were determined by flow cytometry using H2-DCF-DA. B16F10 cells (1 × 10^6^/mL) were incubated at 37 °C in a 5% CO_2_ incubator. The next day, 2 × 10^9^ cells were infected with *S. Typhimurium* expressing miRNA at an MOI of 1:1000 (B16F10 cell: *Salmonella*). Six hours later, the cells were irradiated with 8 Gy. Then, 100 μg/mL gentamicin (Invitrogen) was added to the cells, and the cells were incubated at 37 °C in a 5% CO_2_ incubator. Two days later, the cells were incubated with H2-DCF-DA (10 μM) for 5 min at 37 °C. The cells were then washed with cold PBS and trypsinized. The trypsinized B16F10 cells were sent for FACS analysis to analyze the fluorescence intensity at 488 nm excitation and 530 nm emission.

### 2.8. Combined Treatment with S. Typhimurium and Irradiation

Mice were subcutaneously (s.c.) inoculated with 100 uL of 1 × 10^8^ colony-forming units (CFUs)/mouse *S. Typhimurium* or Recombinant *S. Typhimurium* suspension. One day after subcutaneous inoculation, the mice were irradiated with γ-rays (3 to 8 Gy). Combined treatment was performed at day 1 with *S. Typhimurium* and at day 2 with irradiation. Control animals were treated only with an s.c. injection of PBS only.

### 2.9. Melanoma Cancer Challenge in Mice

For tumor implantation, 6–7-week-old female C57BL6 (n = 10) mice were subcutaneously injected on the mid-right side with B16F10 (1.5 × 10^5^) melanoma cells in 100 μL of PBS. The tumors were allowed to grow for 8−10 days before subsequent treatment. When tumor volumes reached ~50−70 mm^3^, the mice were pooled and randomly assigned to the following four groups: PBS, unmodified *S. Typhimurium*, *S. Typhimurium* expressing control miRNA (*S. Typhimurium*/mi-Cont), and *S. Typhimurium* expressing INHA miRNA (*S. Typhimurium*/mi-INHA). The anti-tumor activity of the genetically engineered *S. Typhimurium* was evaluated by measuring tumor growth inhibition. Tumor volumes were measured every 7 days and calculated as length × width^2^ × 0.52. The overall condition of the mice, including appearance, posture, behavior, and physiological responses, was examined at least three times per week and documented until signs of morbidity and mortality were observed. Mice were deemed cured if they had no visible or palpable tumors for at least 9 (for B16F10) weeks after treatment. The survival of each mouse was recorded, and overall survival was calculated. This study was conducted in accordance with the animal testing guidelines of the Institutional Animal Care and Woojung Bio. IACUC (IACUC 2101-024).

### 2.10. Bacterial Distribution and Cytokine Analysis in Mice

Mice treated with miRNA-expressing bacteria were sacrificed and each tissue was excised for bacterial distribution. Part of the excised tissue was used to count the remaining recombinant bacteria, which were plated on LB agar containing spectinomycin, and the colony count was determined on the next day. A mouse model was subcutaneously injected with 1 × 10^8^ of the recombinant *Salmonella* strains. Blood was also collected from all mice 1 day before and 5 days after subcutaneous administration. Blood samples were stored at 5 °C for 12 h. After coagulation, sera were collected by centrifugation (5 min, 2000× *g* at 4 °C). At the end of the experiment, samples of the same group that had been collected at the same time were pooled and tested using IFN-γ sandwich ELISA kits (Bender Medsystems Inc., CA, USA) with triplicate wells.

### 2.11. Statistical Analysis

All data are expressed as mean ± standard deviation (SD) and compared for statistical significance using the Student’s t-test. Mouse survival experiments were analyzed using Kaplan–Meier survival curves and log-rank test. *p*-values in figures are indicated with asterisks (*; *p* < 0.05, **; *p* < 0.01, ***; *p* < 0.001).

## 3. Results

### 3.1. Application of Bacterial Treatments to Radiotherapy

**Figure 1:**
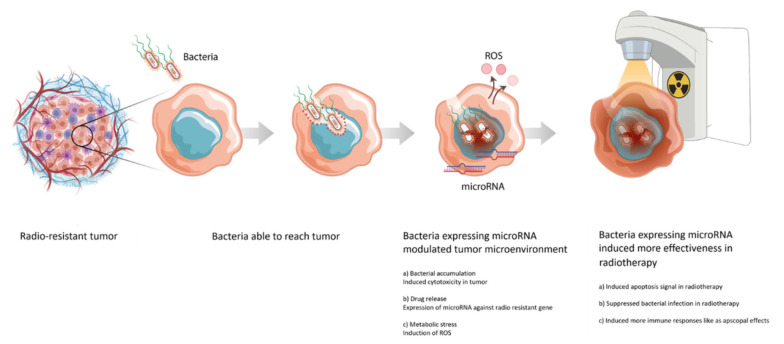
Application of bacterial treatment for combined radiation therapy.

The complex radiation therapy model using bacteria was designed as follows. At first, to suppress the INHA gene that is overexpressed in radiation-resistant cancers, we constructed the *Salmonella* strains expressing the INHA miRNA operated by the *Salmonella* promoter. The constructed *Salmonella* strains could produce the INHA-specific miRNA. In this study, miRNAs were designed to be delivered using *Salmonella* strains to effectively target cancer cells. In particular, a system was designed for delivering miRNA using the strain to target cancer cells through a prokaryotic expression system. This was performed by improving the previously developed vector for INHA delivery and could be effectively applied in combination therapy with radiation (Figure 1).

### 3.2. Construction of Recombinant Salmonella Expressing miRNA for Radioresistant Gene

In a previous study, we confirmed the expression levels of INHA in radioresistant melanoma [6]. We found that its expression level in mouse melanoma tissue was higher than that in normal skin tissue. These results indicate that INHA might be associated with melanoma cancer growth.

**Figure 2:**
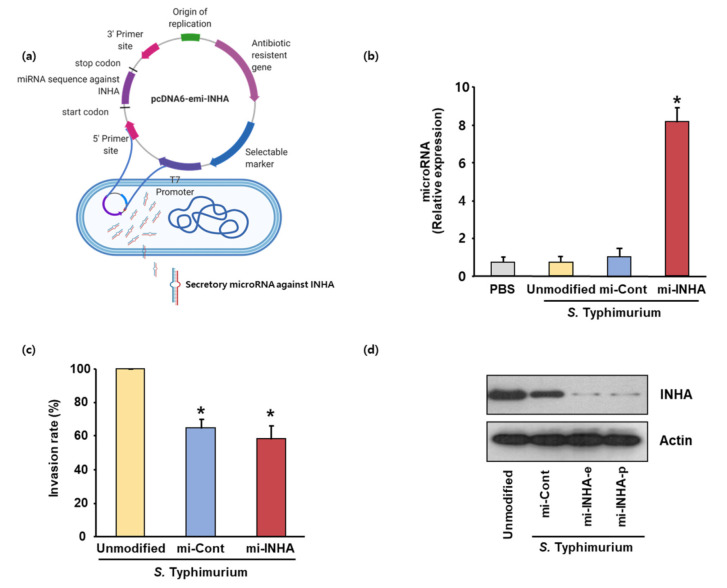
Construction of *S. Typhimurium* expressing microRNA against INHA gene. (**a**) Schematic representation of modified pcDNA6.2-GW/EmGFP-miR vector expressing the mi-INHA. (**b**) To validate the production of mi-INHA, the expression of microRNA was measured in bacterial cells. MicroRNA from genetically engineered *S. Typhimurium* were analyzed using RT-qPCR. (**c**) B16F10 mouse melanoma cells were infected with the indicated *S. Typhimurium* at a multiplicity of infection (MOI) of 500 for 1 h (for invasion assay). The invasion rate of mouse melanoma cells by the unmodified *S. Typhimurium* was set to 100%, and the relative internalization levels were normalized against those of unmodified *S. Typhimurium*. The data represent the mean ± SEM of three independent experiments (* *p* < 0.05 vs. unmodified *S. Typhimurium*-treated samples; Student’s *t*-test). (**d**) B16F10 cells were infected with unmodified *S. Typhimurium* or genetically modified *S. Typhimurium* expressing negative control mi-RNA or mi-INHA for 48 h. To validate INHA knockdown in cancer cells after infection with recombinant *Salmonella*, the expression of mouse INHA was measured. Western blot normalized to β-actin was added as supplementary material (Supplementary Figure S1). The cell lysates were subjected to Western blot analysis with anti-INHA or anti-actin antibodies.

To effectively prevent INHA expression in melanoma cells during radiotherapy, we used an miRNA tool. To construct the INHA miRNA vector for RNA interference (RNAi), two single-stranded DNA oligonucleotides encoding INHA-targeting pre-miRNAs were designed using an RNAi design program (Invitrogen). To enhance the expression efficiency of miRNA in melanoma cancer cells, two single-stranded oligos were annealed and cloned into a modified pcDNATM6.2-GW/miR vector without a CMV promoter (Figure 2a). The constructed vector was transformed into attenuated *Salmonella Typhimurium*. Finally, we constructed an attenuated *S. Typhimurium* BRD509 strain expressing INHA miRNA. To confirm whether the constructed plasmid was expressed in prokaryotic cells, we extracted whole RNA from the constructed *S. Typhimurium*. As shown in Figure 2b, INHA expression was observed in the transformed *S. Typhimurium* (INHA miRNA) but not in the *S. Typhimurium* control.

To assess the bacterial invasion of melanoma cancer cells, a gentamycin protection assay was performed. The ability of *Salmonella* to infect B16F10 cells was evaluated in vitro using attenuated *S. Typhimurium* BRD509 strains expressing the INHA miRNA vector and control vector. Attenuated *S. Typhimurium* strains expressing the INHA miRNA vector were capable of infecting B16F10 cells (Figure 2c).

To investigate whether the INHA is downregulated in melanoma cells, whole protein was extracted from melanoma cells infected with constructed *S. Typhimurium*. Proteins were analyzed using Western blotting. Western blot analysis showed that INHA expression was only downregulated in melanoma cells infected with *S. Typhimurium* expressing the INHA miRNA vector (Figure 2d).

### 3.3. Reduction in Tumor Cell Viability by S. Typhimurium Expressing miRNA-INHA with Irradiation

We next examined the combined effect of attenuated *S. Typhimurium* expressing the INHA miRNA vector and radiation on melanoma models, and applied irradiation (8 Gy) to the LDH assay and apoptosis assay.

**Figure 3:**
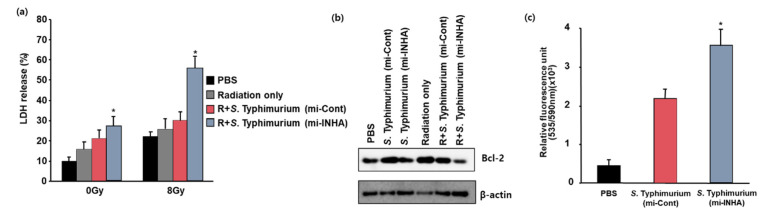
Combinatory effects of *S. Typhimurium* expressing mi-INHA and radiation in B16F10 cells. (**a**) LDH cytotoxicity assays were performed on cell-free supernatants from B16F10 cells. Cytotoxicity was determined by measuring the amount of LDH released from PBS-treated cells or *S. Typhimurium*-treated cells into the supernatant with respect to cells exposed to a detergent. Each data point represents the mean ± SEM of three independent experiments (* *p* < 0.05 vs. PBS-treated controls; Student’s *t*-test). (**b**) Western blotting was used to detect the expression patterns of Bcl-2, b-actin, and INHA. The blots are representative of three independent experiments. Western blot normalized to β-actin was added as supplementary material (Supplementary Figure S2). (**c**) ROS production was measured by FACS analysis in B16F10 cells using 10 μM of H2DCFDA, which converts to a fluorescent derivative only in the presence of ROS. Flow cytometry histogram data were added as supplementary material (Supplementary Figure S3). Results shown are representative of at least three independent experiments.

Melanoma cell death was evaluated by measuring the LDH release into the media from dead or dying cells at 24 and 48 h after the *Salmonella* infection. There was a slight difference in cell lysis 24 h after infection (data not shown). However, at 48 h after infection, there was a clear difference in melanoma cell lysis between *S. Typhimurium* expressing the INHA miRNA vector and the control vector. At 48 h after infection, the LDH release increased by 32% in the supernatant of melanoma cells infected with *S. Typhimurium* expressing INHA miRNA upon irradiation, compared to the control group (Figure 3a). The amount of LDH released from melanoma cell lysis was >two-fold higher than that with the control vector at 48 h after infection. These results indicated that the attenuated *S. Typhimurium* expressing INHA miRNA could suppress melanoma cell growth and induce efficient cell death.

To confirm whether the constructed *Salmonella* induces cell death through the apoptosis pathway in melanoma cells, we investigated the expression levels of the anti-apoptotic protein Bcl-2 in infected melanoma cells. Western blot analysis (Figure 3b) showed that the expression level of Bcl-2 decreased in infected melanoma cells.

To further confirm this result, we investigated reactive oxygen species (ROS) in melanoma cells infected with the constructed *Salmonella* strains expressing miRNA (INHA miRNA vector and control vector). Intracellular hydroperoxide levels were determined by flow cytometry using H2-DCF-DA. As shown in Figure 3c, ROS were significantly increased in the melanoma cells infected with the constructed *Salmonella* strains expressing INHA miRNA when compared to those in the PBS control and the control vector-harboring *Salmonella* groups. Likewise, the quantified relative ROS increased in the melanoma cells infected with the constructed *Salmonella* strain expressing the INHA miRNA vector. These results show that the INHA downregulation in melanoma cells increases ROS levels and downregulates the expression of Bcl2, suggesting that the constructed *Salmonella* activates the apoptosis pathway and that ROS levels are significantly increased in infected melanoma cells.

### 3.4. Effects of Combined Treatments with S. Typhimurium Expressing miRNA and Irradiation in Animals Bearing Melanoma

C57BL/6 mice were subcutaneously injected with B16F10 melanoma cells (1 × 10^6^). Mice were inoculated with approximately 1 × 10^9^ CFU of attenuated *S. Typhimurium* expressing miRNA (INHA miRNA, Control vector) via subcutaneous injection 2 weeks after melanoma cell challenge. Tumor growth was then monitored in the mice. The melanoma tumor diameter was measured using a digital caliper. Further, the survival rates of the mice were monitored.

**Figure 4:**
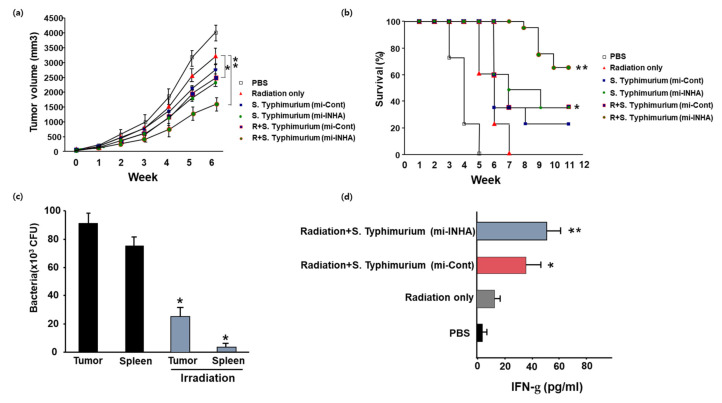
Effects of combined bacterial therapy with radiation in mice bearing melanoma. C57BL/6 mice bearing were inoculated s.c. on days 1 with PBS or with 1 × 10^8^ CFU of attenuated unmodified *S. Typhimurium*, *S. Typhimurium*/mi-Cont, or *S. Typhimurium*/mi-INHA. After s.c. inoculation of bacterial cells. The mice were exposed to γ-radiation (8 Gy) on day 2 (**a**) The tumor volume was measured for up to 6 weeks after combined treatment with bacterial cells and irradiation. Data are presented as the mean ± SEM of three independent experiments. (**b**) The survival rates of all groups were recorded for up to 12 weeks. The data are presented as Kaplan–Meier survival curves, and comparisons were made using the log-rank test. (**c**) Bacterial distribution was examined in mice after s.c. inoculations with 1 × 10^7^ bacterial cells. At 5 days, bacteria were not cultivated from tissue in mice with subcutaneous recombinant *Salmonella* inoculation. (**d**) Interferon gamma expressions were induced in mice treated with *S. Typhimurium* expressing IFN-g. Mice were treated with PBS and 1 × 10^8^ bacterial groups. Serum was collected 7 days after inoculation and were analyzed with ELISA. *p*-values in figures are indicated with asterisks (*; *p* < 0.05, **; *p* < 0.01).

To examine the combined effect of attenuated *S. Typhimurium* expressing INHA miRNA and radiation on melanoma models, we additionally applied irradiation (8 Gy) to animals using the melanoma model. Figure 4a,b show that tumor sizes decreased, and survival rates increased in the group treated with the combination therapy compared to those in the group treated with only attenuated *S. Typhimurium* expressing INHA miRNA. These results show that the anticancer effect was enhanced in the group treated with radiation and attenuated *S. Typhimurium* expressing the INHA miRNA vector, with combined therapy exhibiting a slightly synergistic effect.

We next investigated the distribution of residual microorganisms in animals during combination therapy with radiation. For this skin cancer mouse model, bacteria were found to be removed from cancer tissues, the liver, and the spleen, among other tissues, within 3–7 days, but when radiation was also used, the strain was removed within 2 days (Figure 4c). In addition, the expression of IFN-γ in animals was increased after combination therapy (Figure 4d).

## 4. Discussion

Cancer is a multicellular disease characterized by uncontrolled cell growth and metastasis. It remains a leading cause of death globally [20,27,28,29]. Over the past few decades, our knowledge about cancer and treatment methods have progressed considerably, together with advances in early diagnosis and treatment modalities such as radiation therapy, chemotherapy, and combination therapy of both radiotherapy and chemotherapy. The recent reports are that INHA miRNA promotes the apoptosis via the mitochondrial pathway, which is followed by leading to the increase in the intracellular ROS level by the mitochondrial membrane depolarization. On the basis of these observations, we designed the experiment combined with the INHA miRNA-harboring *Salmonella* and the irradiation for overcoming the limitation of cancer treatment. With the invasion assay, *Salmonella* expressing INHA miRNA showed that the generation of intracellular ROS is increased, and the expression of Bcl-2 is suppressed in the protein level (Figure 2 and Figure 3). On the other hand, it is reported that the intracellular ROS could be generated when the irradiation is exposed to the cancer cells [30]. Using these observations, we tried to examine whether or not the level of intracellular ROS induced by INHA miRNA is related with the irradiation. As shown in Figure 4, the combined treatment with INHA-bearing *Salmonella* and irradiation showed an anticancer effect.

Recently, the abscopal effect, which was used by Mole in 1953, has gained the attention of researchers with the advent of immunotherapy [31,32]. Over the years, it has been reported that cancer cells are monitored by the immune system, and that they have a variety of mechanisms that allow them to evade the host immune system [33,34]. That means that it does not invoke the appropriate immune responses, diminishing the body’s ability to remove aberrant tumor cells. Radiation therapy stimulates latent immune cells and is a well-known treatment for cancer, with more than 50% of newly diagnosed cancer patients with solid tumors receiving radiation as treatment. Its mechanism involves causing damage to the DNA backbone or the generation of ROS [16]. In addition, it has been reported that radiation influences the expression of cytokines in the body, such as TNF-alpha and interleukin-1 (IL-1) [33]. However, the molecular mechanism of radiation-induced cell death remains to be elucidated. The activation of immune responses by radiation could suppress distant cancer cells, mitigating the effect of metastasis. Therefore, immunotherapy and radiotherapy could exhibit synergistic effects. Therefore, we attempted to show that a *Salmonella* vector with miRNA could enhance the antitumor effects of radiation therapy.

Cancer therapy using *Salmonella* vectors has been intensively studied for decades [4,23,35,36]. *Salmonella* has tumor-targeting properties, can reactivate the anergy state of local immune cells within tumors, suppress Treg cells within the tumor, and can be used as a delivery vector with effector molecules (proteins and genetic materials) or miRNA. However, there are concerns about the instability of the *Salmonella* vectors. *Salmonella* is a complex genus that replicates and can invoke immune responses in humans [37].

Recently, it has been suggested that the microbiome might be a component of the immune system [38]. In general, the immune system maintains a germ-free state in the host. However, germ-free mice require dietary supplementation with microbial products (e.g., vitamin K) and are highly sensitive to infection [38], which means that the immune system utilizes the microbiome as an adjuvant. Additionally, it has been reported that short-chain fatty acids metabolized by the gut microbiome impair antigen-presenting cells [37,39]. When *Salmonella* is introduced into the gut microbiota, the gut environment shifts from homeostasis to dysbiosis. Using an engineered *Salmonella* vector combined with radiotherapy, we showed that *Salmonella* strains expressing miRNAs are associated with the production of ROS, which is involved in radiation sensitivity, and have been found to increase treatment efficiency when combined with radiation therapy. Interferon gamma induced by recombinant *Salmonella* is thought to enhance the abscopal effect [32], suggesting the possibility of radiation immunotherapy using *Salmonella* strains. In addition to the attenuation of the strain itself, an advantage in terms of safety can be expected with the sterilization effect [40], in which more than 80% of bacteria are removed by radiation. In conclusion, as an effective treatment strategy for refractory cancer, *Salmonella* strains that secrete miRNAs and suppress cancer-specific gene overexpression are effective. In particular, when combined with microRNA-expressing *Salmonella* and radiotherapy, the enhancing radiation therapy efficacy can be expected.

## Supplementary Materials

The following are available online at https://www.mdpi.com/article/10.3390/microorganisms9112408/s1, Figure S1: Suppression of INHA gene by *S. Typhimurium* expressing microRNA against INHA gene B. Figure S2 Combinatory effects of *S. Typhimurium* expressing mi INHA and radiation in B 16 F 10 cells. Figure S3. Combinatory effects of *S. Typhimurium* expressing mi-INHA and radiation in B16F10 cells.
