# Supplementary material for: Combined Therapy with microRNA-Expressing Salmonella and Irradiation in Melanoma
Source: Microorganisms. 2021 Nov 22;9(11):2408. doi: 10.3390/microorganisms9112408 (PMC8621892; doi:10.3390/microorganisms9112408)
Supplement: Supplementary file 1 [file microorganisms-09-02408-s001.zip › microorganisms-1440244-supplementary.pdf]

Supplementary Figure S1.

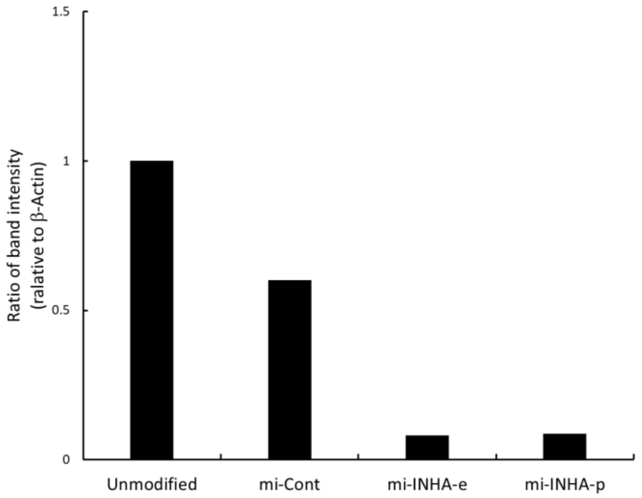

Supplementary Figure S1. Suppression of INHA gene by S. Typhimurium expressing microRNA against INHA gene. B16F10 cells were infected with unmodified S. Typhimurium or genetically modified S. Typhimurium expressing negative control mi-RNA or mi-INHA for 48 h. To vali-date INHA knockdown in cancer cells after infection with recombinant *Salmonella*, the expression of mouse INHA was measured. Western blot normalized to β-actin was added as supplementary material (Supplementary Figure S1). The cell lysates were subjected to Western blot analysis with anti-INHA or anti-actin antibodies.

Supplementary Figure S2.

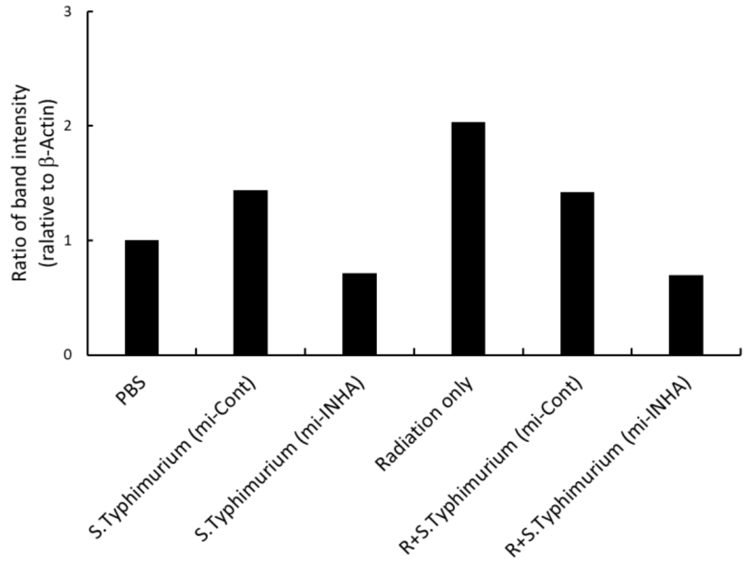

Figure S2. Combinatory effects of S. Typhimurium expressing mi-INHA and radiation in B16F10 cells. Western blotting was used to detect the expression patterns of Bcl-2, b-actin, and INHA. The blots are representative of three independent experiments. Western blot normalized to β-actin was added as supplementary material (Supplementary Figure S2).

Supplementary Figure S3.

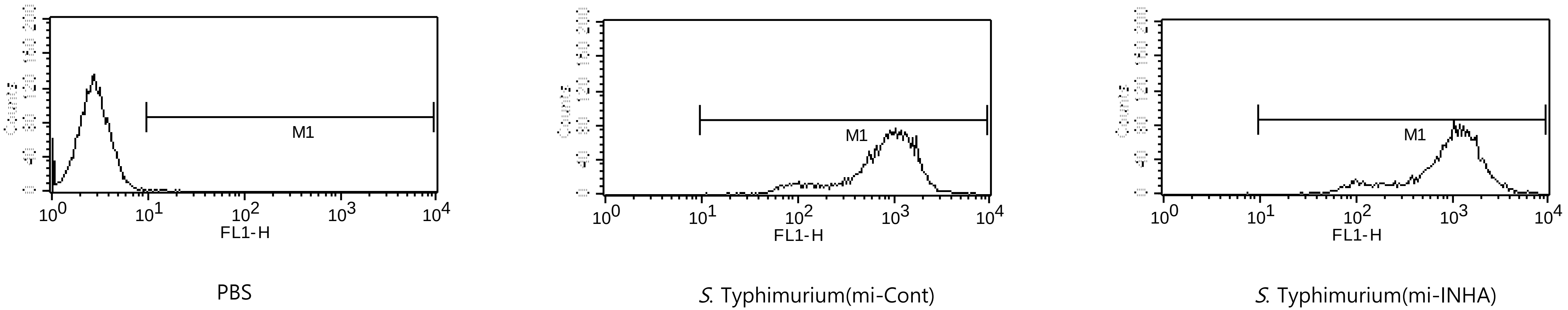

Figure S3. Combinatory effects of S. Typhimurium expressing mi-INHA and radiation in B16F10 cells. ROS production was measured by FACS analysis in B16F10 cells using 10 μM of H2DCFDA, which converts to a fluorescent derivative only in the presence of ROS. Flow cytometry histogram data was added as supplementary material (Supplementary Figure S3). Results shown are representative of at least three independent experiments.
